# A pragmatic single-group evaluation of a self-determination theory-informed health literacy course for exercise behavior readiness among Japanese university students

**DOI:** 10.3389/fspor.2026.1821452

**Published:** 2026-07-15

**Authors:** Issei Ogasawara, Shoji Konda, Susumu Iwasaki, Kayo Yoda-Tsumura, Tatsuya Hayashi, Tomoyuki Matsuo, Takufumi Yanagisawa, Ken Nakata

**Affiliations:** 1 Neuroinformatics, Department of Health and Sport Sciences, Graduate School of Medicine, The University of Osaka, Toyonaka, Japan; 2 Department of Health and Human Performance, Fort Lewis College, Durango, CO, United States; 3 Medicine for Sports and Performing Arts, Department of Health and Sport Sciences, Graduate School of Medicine, The University of Osaka, Suita, Japan

**Keywords:** affective response, health literacy, physical activity, self-determination theory, university students

## Abstract

**Introduction:**

Health literacy education may provide a pragmatic setting for supporting students’ reflection on physical activity and health behaviors when embedded within credit-bearing university courses. However, evidence from course-integrated, single-group evaluations remains limited. This exploratory study examined changes in exercise behavior readiness and related process indicators during a 15-week “Smart Health Literacy” course at The University of Osaka.

**Methods:**

Forty-four first-year students engaged in weekly self-directed goal setting, inclusive physical activities, and data-driven instruction using wearable sensors. Assessments included pre- and post-course stages of exercise behavior change, weekly self-rated goal-attainment effort, free-living step counts, and session-specific affective responses during a cooperative Mölkky session. The primary outcome was analyzed using a mixed-effects ordinal regression model with participant-specific random intercepts.

**Results:**

The mixed-effects ordinal regression model indicated that the post-course assessment was associated with higher exercise behavior stages compared with baseline (OR=3.89, 95% CI: 2.57–5.89; Wald-type *p* < 0.001). Weekly goal-attainment effort increased significantly over the semester, reflecting greater self-rated effort toward self-selected health goals. The Mölkky session elicited acute changes in both positive and negative affect among PANAS complete cases (*n* = 26). In contrast, objectively measured step counts significantly decreased from early to late semester, indicating that free-living ambulatory activity did not improve during the observation period.

**Discussion:**

These findings provide preliminary evidence that a course-based health literacy program may be associated with increased exercise behavior readiness and self-regulatory engagement with self-selected health goals. However, because this was a single-group pre–post evaluation without a control group, the findings should be interpreted as exploratory associations rather than evidence of causal intervention effectiveness.

## 1 Introduction

Regular physical activity (PA) is a fundamental health behavior for the prevention of noncommunicable diseases across the life course. The World Health Organization recommends that adults, including university-aged young adults, should accumulate at least 150–300 min/week of moderate-intensity aerobic activity, 75–150 min/week of vigorous-intensity activity, or an equivalent combination, while reducing sedentary time (1). However, despite these guidelines, nearly one-third of adults worldwide (31%) did not meet these recommendations in 2022—a proportion that has worsened since 2010 (2). If current trends continue, global physical inactivity is projected to increase further by 2030, underscoring the urgency for scalable solutions that are feasible in real-world settings (2).

In Japan, insufficient PA is particularly concerning among young adults. According to the 2019 National Health and Nutrition Survey, the proportion of individuals reporting a “regular exercise habit” (≥ 30 min per session, ≥ 2 sessions/week, sustained ≥ 1 year) was low in younger age groups: 12.9% of women and 28.4% of men aged 20–29 years, with women aged 30–39 years recording the lowest prevalence at 9.4% (3). Objectively measured ambulatory activity also showed a decade-long decline among women and relatively stagnant levels among men. In 2019, mean daily step counts were approximately 6,641 for women and 8,301 for men aged 20–29 years. Collectively, these patterns indicate both inadequate participation and unfavorable trends throughout late adolescence and emerging adulthood.

The transition from high school to university is a pivotal period during which PA commonly declines, shaped by new schedules, social demands, and changing environments. Longitudinal evidence and systematic reviews document reductions in moderate-to-vigorous PA during this transition, highlighting time management, academic pressure, and psychosocial factors as key constraints (4). From a public-health and pedagogical standpoint, however, this “window of risk” is also a “window of opportunity”: nearly all students can be reached via credit-bearing, semester-long courses (5). Thoughtfully designed first-year curricula may therefore function as a structural lever for shifting PA-related attitudes, motivations, and behaviors at scale.

One promising approach is health literacy (HL)-oriented instruction that blends conceptual knowledge with experiential practice. HL—encompassing functional, interactive, and critical competencies—can empower learners to find, interpret, and apply health information and to participate more effectively in health-promoting practices (6). Recent studies indicate that HL interventions implemented in higher education settings can improve a range of health outcomes, including PA, sleep quality, and mental well-being, especially when they employ interactive and student-centered strategies (7).

Behavior change theories further clarify how course design might facilitate meaningful PA adoption. For example, the transtheoretical model conceptualizes adoption and maintenance as progressive movement through stages of readiness (i.e., pre-contemplation, contemplation, preparation, action, and maintenance), with the maintenance stage defined by sustained regular activity for at least six months (8, 9). Applications of this model among university students and Japanese populations support the validity of these stage constructs and their associations with psychosocial determinants, such as self-efficacy (10, 11). However, the six-month maintenance criterion complicates evaluation within a single 15-week university semester in Japan.

Self-determination theory (SDT) offers a complementary framework, positing that learning designs that support autonomy, competence, and relatedness promote higher-quality, more autonomous motivation and greater long-term adherence (12, 13). Meta-analytic evidence has shown that SDT-based instructional strategies can beneficially shape motivation and engagement in both PA and educational contexts (14, 15). Alongside motivation, the affective experience of activity—how individuals feel during and immediately after exercise—has emerged as a robust predictor of future participation in PA (16–18). Thus, course designs that deliberately elicit enjoyable, socially connected activity and help students track and reflect on their experiences may support readiness for behavior change and self-regulatory engagement.

Universities also offer a pragmatic setting for data-driven pedagogy. Incorporating wearable devices, simple physiological tasks, and student-led data analysis can make PA visible and personally meaningful while simultaneously building data and HL (19). Emerging reviews on wearable technology and biosensor use in education have highlighted their value for engagement, real-time feedback, and reflective learning when integrated with sound pedagogical frameworks (20, 21). Such practice-embedded approaches align with calls for pragmatic PA intervention research.

Building on this context, the present study conducted a pragmatic, exploratory evaluation of a semester-long undergraduate HL course that integrated SDT-informed pedagogical design, weekly goal setting and reflection, diverse and inclusive PA experiences, and data exercises using wearable sensors and simple laboratory tasks. We examined whether students were more likely to be classified in higher stages of exercise behavior readiness after the course. We also evaluated weekly self-rated effort toward self-selected health goals, free-living step counts, and session-specific affective responses to a cooperative group activity as secondary or process-related outcomes. We hypothesized that, even within a single semester, students would show a favorable shift toward higher readiness for regular exercise, accompanied by increases in goal-directed effort and daily free-living step counts. Given the single-group design, the study was intended to generate preliminary evidence regarding feasibility and within-participant change rather than to establish causal intervention effectiveness.

## 2 Methods

### 2.1 Study period

This study was conducted from April 10 to October 13, 2025. The course-based intervention was implemented from April 10 to July 29, 2025. After final course grades were confirmed, a two-week opt-out period commenced on September 28, 2025, and the dataset was finalized on October 13, 2025.

### 2.2 Participants and recruitment

Participants were 44 first-year university students enrolled in the first author's “Smart Health Literacy” course (women: *n* = 17; mean height 158.2 ± 3.9 cm; mean weight 50.2 ± 6.4 kg; mean age 18.4 ± 0.6 years; age range 18–20 years; men: *n* = 27; mean height 173.4 ± 4.5 cm; mean weight 63.5 ± 7.9 kg; mean age 18.3 ± 0.6 years; age range 18–20 years). The course was one of several concurrently offered required credit-bearing courses, and students were assigned to one of these courses through the university's lottery-based allocation system. Thus, although students did not voluntarily select this specific course, they were required to complete the assigned course activities to receive credit.

Informed consent for research use of course data was obtained using an opt-out procedure approved by the Institutional Ethics Review Committee of The University of Osaka Hospital (approval number: 19537-10). Because the study was conducted within a credit-bearing course, we carefully distinguished between activities conducted for educational purposes and the voluntary use of course data for research.

During Session 2, students were informed that the course included measurement, data analysis, and interpretation activities as part of its educational objectives. These activities included wearable sensor-based measurements, questionnaire-based reflection, and analysis of physical and psychological data. Students assigned to the course could not decline the educational components of the course if they wished to receive credit, because measurement, reflection, data analysis, and portfolio assignments were part of the course learning objectives and assessment. However, research participation was limited to the later use of course data for research purposes and was separate from course completion. Students could decline the research use of all or part of their course data. Such refusal had no effect on course grades, credit acquisition, or any future university activities.

During the course, personal identifiers attached to the course data were replaced with student-specific IDs. Students used these ID-coded data when practicing data analysis as part of the course activities. Because instructors were responsible for individual course assessment, a correspondence table linking student IDs to individual students was maintained during the course. Access to this correspondence table was restricted to the course instructors. Research analyses were conducted only after final grades had been confirmed and the opt-out period had ended.

Students were told that research use of course data would occur only after the course had ended and final grades had been finalized. They were informed that they could decline the use of all or part of their data for research and that such refusal would have no effect on their course grade, credit acquisition, or any future university activities. They were also informed that, once aggregated results had been published, it would not be possible to remove their data from published outputs.

These points were explained again orally during the final class session (29 July 2025). After final grades had been confirmed and could no longer be changed, all students received an opt-out email on September 28, 2025, through the university's educational ICT system. The email reiterated the study purpose, the voluntary nature of research participation, the right to refuse use of all or part of their course data, and the absence of any disadvantage associated with refusal. Students were given a two-week opt-out period. No students requested withdrawal of their data, and the final research dataset was fixed on October 13, 2025.

The consent procedure was designed to protect voluntariness in this required educational setting by clearly distinguishing the educational use of course data from the subsequent research use of those data. The collection, visualization, and interpretation of course data were first and foremost educational activities intended to strengthen students’ health-literacy-related knowledge, practical judgment, and reflective understanding of physical and psychological data. Accordingly, students assigned to the course were required to complete these educational activities and portfolio assignments to receive credit. However, research participation was limited to the later use of course data for research purposes, and students could decline the research use of all or part of their data.

To structurally separate course assessment from research participation, the opt-out procedure was implemented only after final grades had been confirmed and could no longer be changed. Therefore, students’ decisions regarding research use of their data could not influence course grading, credit acquisition, or subsequent educational opportunities. This procedure was developed in consultation with, and approved by, the Institutional Ethics Review Committee of The University of Osaka Hospital. It was also consistent with ethical guidance emphasizing transparency, voluntariness, the right to withdraw, and protection from undue influence in educational research (22, 23).

### 2.3 Intervention

#### 2.3.1 SDT-informed course design

The course was designed to foster an autonomy-supportive learning environment in line with SDT principles. Professors, who had prior familiarity with SDT through professional development opportunities, were attentive to students’ perspectives, explained the rationale behind the activities rather than simply forcing participation, and supported their autonomy within the designed course structure.

The intervention was delivered during the spring–summer semester from April 10 to July 29, 2025. Although this period included national holidays in early May, classes were generally held once per week (Tuesdays) for a total of 15 sessions (Table 1). Seasonal conditions were considered in course planning. In Japan, April–May is relatively mild and suitable for outdoor activities; after the rainy season in June, July brings summer conditions, with temperatures often exceeding 30 °C and limiting outdoor activities. To encourage healthy behaviors, we implemented the following instructional strategies.

**Table 1:** Class schedule and data measurement.

Session	Date	Contents	SDT aim	Survey	Measurement	Temperature, weather, location
1	10 April	General online lecture of healthy lifestyle, Risk of smoking and alcohol use, and mental health.				20.4 ℃, Sunny, (Online)
2	22 April	Guidance, Research explanation, Goal settings	Autonomy	Stage of exercise behavior change		24.7 ℃, Sunny, Lecture room
	29 April	National holiday				
3	7 May^a^	Nordic walking in the campus		Goal-attainment (7-Likert)	Step count (sensor hand out)	18.8 ℃, Cloudy, Campus
4	13 May	Blindness simulation experience and guide practice 1	Autonomy, Relatedness	Goal-attainment (7-Likert)	Step count(sensor collection)	25.5 ℃, Sunny, Campus
5	20 May	Blindness simulation experience and guide practice 2	Autonomy, Relatedness	Goal-attainment (7-Likert)		27.6 ℃, Sunny, Campus
6	27 May	Handball	Competence, Relatedness	Goal-attainment (7-Likert)	HR/ACC	24.5 ℃, Sunny, Outdoor field
7	3 June	Ankle taping practice 1	Competence	Goal-attainment (7-Likert)		18.3 ℃, Rainy, Lecture room
8	10 June	Ankle taping practice 2	Competence	Goal-attainment (7-Likert)		21.9 ℃, Rainy, Lecture room
9	17 June	Orienteering in the campus	Autonomy, Competence, Relatedness	Goal-attainment (7-Likert)	HR/ACC	30.5 ℃, Sunny, Campus
10	24 June	Grip strength grading experiment	Competence	Goal-attainment (7-Likert)	HR/ACC	28.0 ℃, Sunny, Lecture room
11	1 July	Sports injury prevention research review (Online)				32.8 ℃, Sunny, (Online)
12	8 July	Mölkky 1	Autonomy, Competence, Relatedness	Goal-attainment (7-Likert)	Step count (sensor hand out)	35.2 ℃, Sunny, Indoor gym
13	15 July	Mölkky 2	Autonomy, Competence, Relatedness	Goal-attainment (7-Likert)	Step count (sensor collection), HR/ACC, PANAS scale	31.9 ℃, Sunny, Indoor gym
14	22 July	Data review, Team building activities	Autonomy, Relatedness	Goal-attainment (7-Likert)		34.0 ℃, Sunny, Lecture room
15	29 July	Summary of class, Data review		Stage of exercise behavior change, Goal-attainment (7-Likert)		36.6 ℃, Sunny, Lecture room

a This session was irregularly conducted on Wednesday.

SDT, Self-determination theory; HR, Heart rate; ACC, Acceleration.

#### 2.3.2 Self-directed goal setting

In Session 2 (April 22), students set personal health goals to pursue during the semester. At the beginning of each subsequent class, they reflected on the previous week and rated their effort toward their goal on a 7-point scale.

#### 2.3.3 Diverse instructional content

Each session offered different instructional content, covering a broad range of experiential and didactic activities, from practical sport experiences to HL knowledge. Experiential components included a competitive team sport (handball), recreational PA (Nordic walking, orienteering, and Mölkky), social inclusion activities (blindness simulation and sighted-guide practice), and a sports medicine skills practicum (ankle taping). Students also participated in a psychophysical motor control experiment (grip strength grading; Supplementary Figure S1-1 in Supplementary 1) and learned how to analyze and interpret PA data. Sessions were conducted across multiple locations—lecture rooms, outdoor fields, indoor gymnasiums, and throughout campus—to maintain novelty and engagement. Full session details are provided in Supplementary 1.

#### 2.3.4 Data-driven instruction

The course incorporated a practical data literacy program in which students learned methods to measure PA during exercise and daily life using wearable sensors, as well as to assess grip strength control in a laboratory setting. Students practiced data visualization and interpretation to support reflective learning and to make PA-related data personally meaningful. This component was designed to be consistent with SDT-informed pedagogy by providing opportunities for autonomy-supportive reflection and competence-oriented learning.

### 2.4 Data collection

The data collection schedule is presented in Table 1.

#### 2.4.1 Stage of exercise behavior change

Exercise behavior change stage was assessed in Sessions 2 and 15 to observe changes over the semester. Assessments distinguished between “lifestyle PA” and “exercise.” Lifestyle PA was defined as activities in daily life (including part-time work) that slightly elevate breathing and heart rate (HR), such as walking (including commuting, dog walking, and shopping), cycling for transportation, domestic tasks, and carrying loads, performed in bouts of ≥ 10 min and totaling ≥ 30 min/day. Exercise was defined as structured sports or exercise, such as running/jogging, swimming, ball sports, and martial arts, that produce noticeably elevated breathing and HR, performed in bouts of ≥ 20 min.

Following Okazaki et al. (24) and Fujita et al. (25), participants selected one of four options (coded 1–4):

1. I am not currently engaging in lifestyle PA or exercise on a regular basis and have no intention of starting in the foreseeable future.
2. I am not currently engaging regularly, but I intend to start.
3. I intend to start soon, or I sometimes engage in lifestyle PA or exercise.
4. I currently engage in lifestyle PA or exercise.

Further sub-classification was made for those who selected option 4:

Lifestyle PA:

4a-1: Not performing lifestyle PA regularly.
4a-2: Lifestyle PA ≤ 4 times/week (≥ 30 min/session).
4a-3: Lifestyle PA ≥ 5 times/week.

Exercise:

4b-1: Not performing exercise regularly.
4b-2: Exercise ≤ 2 times/week (≥ 20 min/session).
4b-3: Exercise ≥ 3 times/week (≥ 20 min/session).

This staging instrument was adapted for Japanese university students’ schedules. In the conventional transtheoretical model, stages above action require at least six months of sustained behavior to qualify as maintenance (8, 26). However, because the present intervention spanned four months, this criterion could not be verified. Therefore, following Fujita et al. (25), stages at and beyond action were classified according to the frequency of lifestyle PA and exercise, rather than the duration of maintenance. Exercise conducted as part of mandatory university physical education classes was excluded from staging. For analysis, these questionnaire responses were mapped to the seven ordered stage categories shown in Table 2.

**Table 2:** Categorization mechanism of the stage of exercise behavior change.

Questionnaire response code	Stage of exercise behavior change
1	Pre-contemplation
2	Contemplation
3	Preparation
4a-1 & 4b-2, 4a-2 & (4b-1 or 4b-2)	Action 1: Both lifestyle PA and exercise are insufficient
4a-3 & (4b-1 or 4b-2)	Action 2: Lifestyle PA is sufficient
(4a-1 or 4a-2) & 4b-3	Action 3: Exercise is sufficient
4a-3 & 4b-3	Action 4: Both lifestyle PA and exercise are sufficient

The classification of stage of exercise behavior change was based on the works of Okazaki et al. (24) and Fujita et al. (25). The codes 4a-1 to 4a-3 and 4b-1 to 4b-3 correspond to increasing frequency levels of lifestyle PA and structured exercise, respectively, as defined in the Methods section.

#### 2.4.2 Goal-attainment effort

At the beginning of each weekly class, students self-rated their effort during the previous week toward their personally set health goal on a 7-point scale (1 = not at all, 4 = neutral, 7 = very much). Students who achieved their initial health goals during the semester defined a new goal and thereafter reported their efforts toward the updated goal (goal updates are presented in Supplementary Table S2 in Supplementary 2).

#### 2.4.3 Free-living step count

Free-living step counts were measured at two time points, each spanning seven consecutive days: early in the semester (May 7–13) and toward the end (July 8–15). Participants wore a waist-mounted wearable sensor (AM550N; SANKA Co., Ltd., Japan) during waking hours, removing it only for sleep and bathing.

#### 2.4.4 Secondary session-specific affective response during the Mölkky session

To assess a secondary, session-specific affective response to a cooperative activity, the Positive and Negative Affect Schedule (PANAS) was administered before and after the Mölkky session (Session 13). The validated Japanese version of the PANAS questionnaire (27) consists of eight positive and eight negative affect items (Table 3), rated on a 6-point Likert scale (1 = “not at all” to 6 = “extremely”) in response to the prompt: “This scale consists of 16 words that describe different feelings and emotions. Read each item and indicate the extent to which you feel this way at present.” Responses were collected at three time points: before the Mölkky class (T1, baseline, 9:00 am), immediately after the session (T2, 10:20 am), and after a subsequent lecture session (T3, follow-up, 12:00 pm). The follow-up lecture session was a foreign-language class (e.g., German or Chinese) conducted by another teacher in a non-group-work format. The questionnaire was administered as a web-based survey completed on participants’ smartphones.

**Table 3:** Positive and negative affect schedule items with Japanese translation. Question sentence: This scale consists of 16 words describing different feelings and emotions. Read each item and indicate the extent to which you feel this way at present. Response: 6-point Likert scale (1 = “Not at all” to 6 = “Extremely”).

Item ID	Item	Japanese translation
PA1	Active	Kakki no aru
PA2	Proud	Hokorashii
PA3	Strong	Tsuyoki na
PA4	Decisive/Determined	Kippari to shita
PA5	Energetic/Spirited	Kiai no haitta
PA6	Excited	Wakuwaku shita
PA7	Agile/Alert/Quick	Kibin na
PA8	Enthusiastic/Passionate	Nekkyo shita
NA1	Jittery/Nervous	Bikubiku shita
NA2	Scared/Afraid	Obieta
NA3	Upset/Distressed	Urotaeta
NA4	Worried	Shimpai shita
NA5	Tense	Piripiri shita
NA6	Distressed/Suffering	Kunou shita
NA7	Ashamed	Hajita
NA8	Irritable	Iradatta

#### 2.4.5 Heart rate and acceleration

HR and acceleration (ACC) were recorded during class sessions using the Polar Verity Sense (Polar Electro, Finland; firmware version 2.2.6), worn on the non-dominant upper arm with a flexible band to ensure continuous optical contact with the skin. Data were recorded continuously from the start to the end of each class and stored in the device's onboard memory. The sampling frequencies were 1 Hz for HR and 50 Hz for ACC. Device control was managed using BPAT Heart Rate software (Sports Sensing Co., Ltd., Japan). Sessions in which HR and ACC were collected are listed in Table 1. These data were used for educational purposes to quantify and visualize the variation in physical load across activity types and were not included as outcome variables to assess intervention effectiveness. See Supplementary 1 Supplementary Figure S1-2 for the results of HR/ACC visualization.

### 2.5 Data analysis

All analyses were conducted in Python 3.9 using scipy.stats (1.13.1), and statsmodels (0.14.5).

#### 2.5.1 Stage of exercise behavior change

Two students did not complete the Session 15 questionnaire; thus, 42 out of 44 responses were included in the analysis. Based on questionnaire responses (code 1–4) at Session 2 and Session 15, the stage of exercise behavior change was categorized into seven ordered levels following Fujita et al. (25): 1: pre-contemplation, 2: contemplation, 3: preparation, 4: Action 1 (insufficient lifestyle PA and exercise), 5: Action 2 (sufficient lifestyle PA only), 6: Action 3 (sufficient exercise only), and 7: Action 4 (both lifestyle PA and exercise are sufficient). Table 2 presents the categorization criteria.

To examine whether participants were more likely to be classified in higher stages at the post-course assessment, we fitted a mixed-effects ordinal regression model using a proportional odds cumulative logit framework. The outcome variable was the stage of exercise behavior change, treated as an ordered categorical variable reflecting increasing readiness for regular exercise. Time point was entered as a fixed effect (0 = pre-course assessment; 1 = post-course assessment), and participant-specific random intercepts were included to account for repeated pre–post observations within individuals.

Because the model was parameterized to estimate the cumulative probability of being at or below each stage threshold, a negative coefficient for time indicated a shift toward higher stages at the post-course assessment. For interpretability, the coefficient for time was converted into an odds ratio representing the odds of being classified in a higher stage at the post-course assessment relative to baseline. Wald-type 95% confidence intervals and *p*-values were calculated from the model estimate and standard error. Statistical significance was set at *p* < 0.05.

#### 2.5.2 Goal-attainment effort

We employed a linear mixed-effects model (LMM) to examine longitudinal changes in goal-attainment effort scores. The 7-point weekly score was treated as the dependent variable, and time (week number) was entered as a fixed effect. To account for within-individual correlations and inter-individual variability in trajectories, we specified each individual as a random intercept and included a random slope for time, allowing the effect of the week to vary across individuals. The model parameters were estimated using restricted maximum likelihood. The primary test of interest was whether the fixed effect of time was significantly positive, indicating an increase in self-rated goal-attainment effort over the semester.

To address the heterogeneity of students’ self-set goals, goals were additionally coded as PA/Ex-related (*n* = 26) or non-PA/Ex-related (*n* = 18). PA/Ex-related goals were defined as goals explicitly referring to walking, running, cycling, exercise, gym training, stretching, or other physical activity. Non-PA/Ex-related goals included body composition/weight-related goals, social/general health goals, and diet/nutrition-related goals (See Supplementary Table S2, Supplementary Material 2). To apply a conservative classification, participants who set multiple goals including both PA/Ex-related and non-PA/Ex-related components were classified into the non-PA/Ex-related category. Sensitivity analyses were then conducted separately by goal type using the same linear mixed-effects modeling approach.

#### 2.5.3 Free-living step count

Because one student lost the sensor, and four did not wear it consistently, data from 39 out of 44 students were included in the analysis. The median daily step count across each seven-day monitoring period was calculated and used as a representative value for each student. Prior to hypothesis testing, the normality of step count distributions and paired differences were examined using the Shapiro–Wilk test. Because no significant deviations from normality were observed (all *p*s > 0.05), a paired-samples *t*-test was conducted to compare mean step counts between the early and late semester periods (*p* < 0.05). The effect size was calculated using Cohen's *d* for paired samples, and 95% CIs for mean differences were computed based on the standard error of the mean difference.

#### 2.5.4 Secondary session-specific affective response during the Mölkky session

The PANAS analysis was conducted as a complete-case analysis. Twenty-six of the 44 students completed the PANAS at all three time points and were included in the analysis. Positive and negative affect scores were calculated as the sum of their respective eight items at each time point. Internal consistency of the Japanese PANAS was evaluated descriptively at each time point using Cronbach's *α* (α = 0.86–0.88; Supplementary Table S3 in Supplementary 3).

To examine session-specific changes in affect, linear mixed-effects models were fitted separately for positive affect and negative affect, with time point as a fixed effect and participant-specific random intercepts included to account for repeated observations within participants. For manuscript-style repeated-measures F statistics, repeated-measures ANOVA was also used for each affect type. *post-hoc* pairwise comparisons among time points were conducted using paired t-tests with Holm correction. Statistical significance was set at *p* < 0.05.

## 3 Results

### 3.1 Stage of exercise behavior change

The mixed-effects ordinal regression model indicated a significant shift toward higher exercise behavior change stages at the post-course assessment. The estimated coefficient for time was negative (*β* = −1.36, SE = 0.21), indicating that the cumulative probability of remaining at or below a lower stage threshold decreased after the course. When expressed as the odds of being classified in a higher stage, the odds ratio for the post-course assessment relative to baseline was 3.89 (95% CI: 2.57–5.89; Wald-type *p* < 0.001). Thus, after accounting for repeated observations within participants, students were more likely to be classified in higher exercise behavior stages at the post-course assessment than at baseline. Given the single-group pre–post design, this result should be interpreted as a within-participant association over time rather than evidence of causal intervention effectiveness.

**Figure 1:**
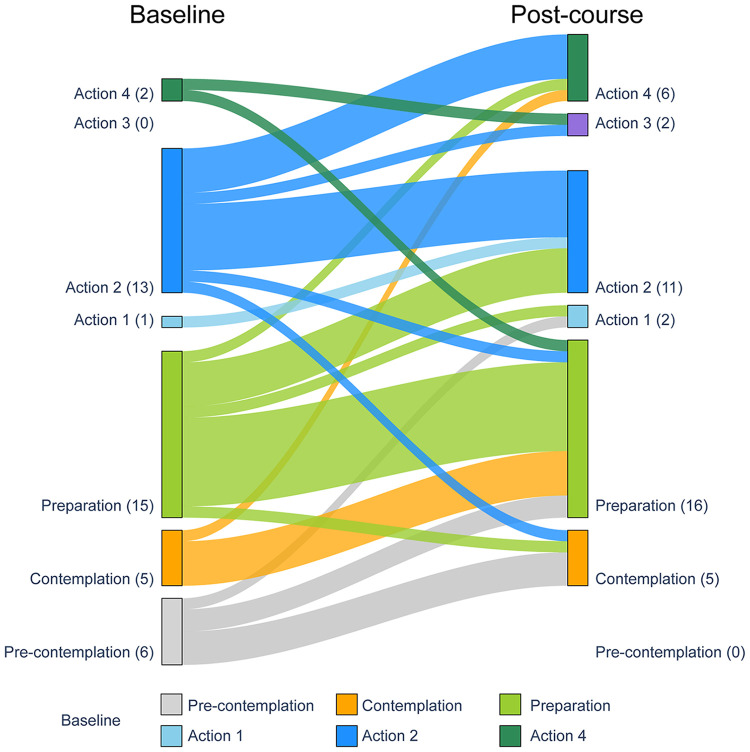
Sankey diagram of exercise behavior change stages from baseline to post-course assessment. All students in the pre-contemplation (*n* = 6) and contemplation (*n* = 5) stages at baseline were classified in higher stages at the post-course assessment.

Before the course, six students were classified into the pre-contemplation stage and five into the contemplation stage; all 11 progressed to either the preparation or action stage after the course. Among the 15 students initially in the preparation stage, six advanced to the action stage, whereas the remaining nine were unchanged. Most students who had already reached the action stage at baseline remained in an action-stage category thereafter (Figure 1).

### 3.2 Goal-attainment effort

**Figure 2:**
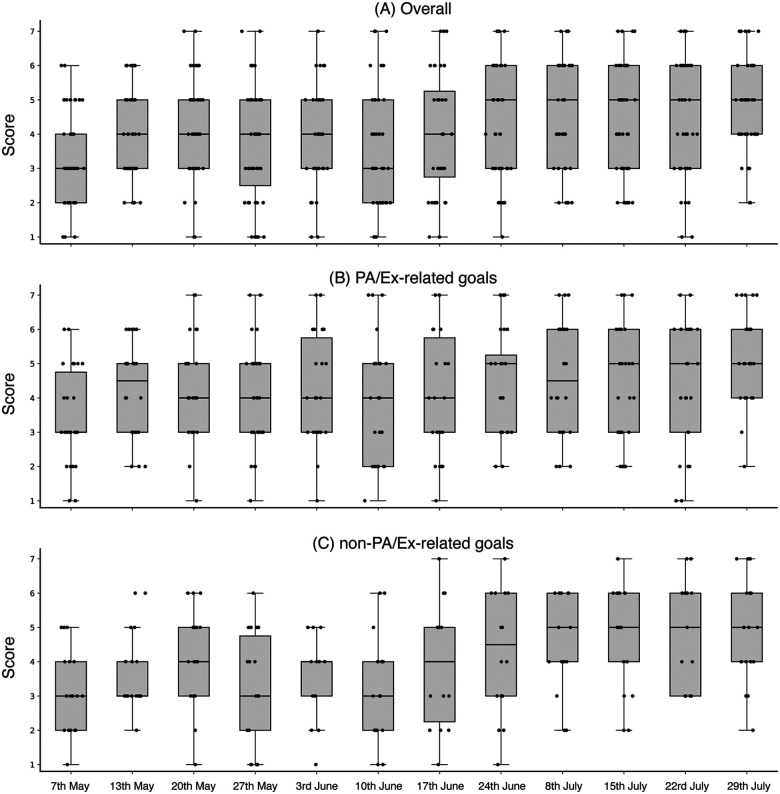
Weekly goal-attainment effort scores over the semester. A linear mixed-effects model revealed a significant positive effect of week number on self-rated goal-attainment effort (*β* = 0.113, SE = 0.023, z = 4.82, *p* < 0.001), indicating increased self-rated effort toward self-selected health goals over the semester.

The LMM analysis revealed a significant positive effect of time on goal-attainment scores (*β* = 0.113, *SE* = 0.023, *z* = 4.82, *p* < 0.001; Figure 2A), corresponding to an average increase of approximately 1.2 points over the semester. Random-effects estimates indicated substantial variability between participants (intercept variance = 1.01; slope variance = 0.012), with a negative covariance (−0.07) suggesting that individuals with lower baseline scores tended to improve more over time. A slope variance of 0.012 indicated moderate inter-individual variability in weekly improvement rates, implying that some participants showed faster progress, while others improved more gradually. Model fit indices showed a log-likelihood of −865.4, an AIC of 1740.8, and a BIC of 1761.7. The marginal *R*^2^ (variance explained by fixed effects alone) was 0.06, whereas the conditional *R*^2^ (variance explained by both fixed and random effects) was 0.42. Time had a small effect size (Cohen's *f*^2^ = 0.06), although it was statistically significant.

In the sensitivity analyses by goal type, the positive time effect on weekly goal-attainment effort remained significant among students with PA/Ex-related goals (*β* = 0.092, SE = 0.030, *p* = 0.002; Figure 2B) and was also observed among those with non-PA/Ex-related goals (*β* = 0.143, SE = 0.037, *p* < 0.001; Figure 2C). These findings indicate that the increase in weekly effort was not limited to exercise-related goals but reflected broader engagement with self-selected health goals.

### 3.3 Free-living step count

**Figure 3:**
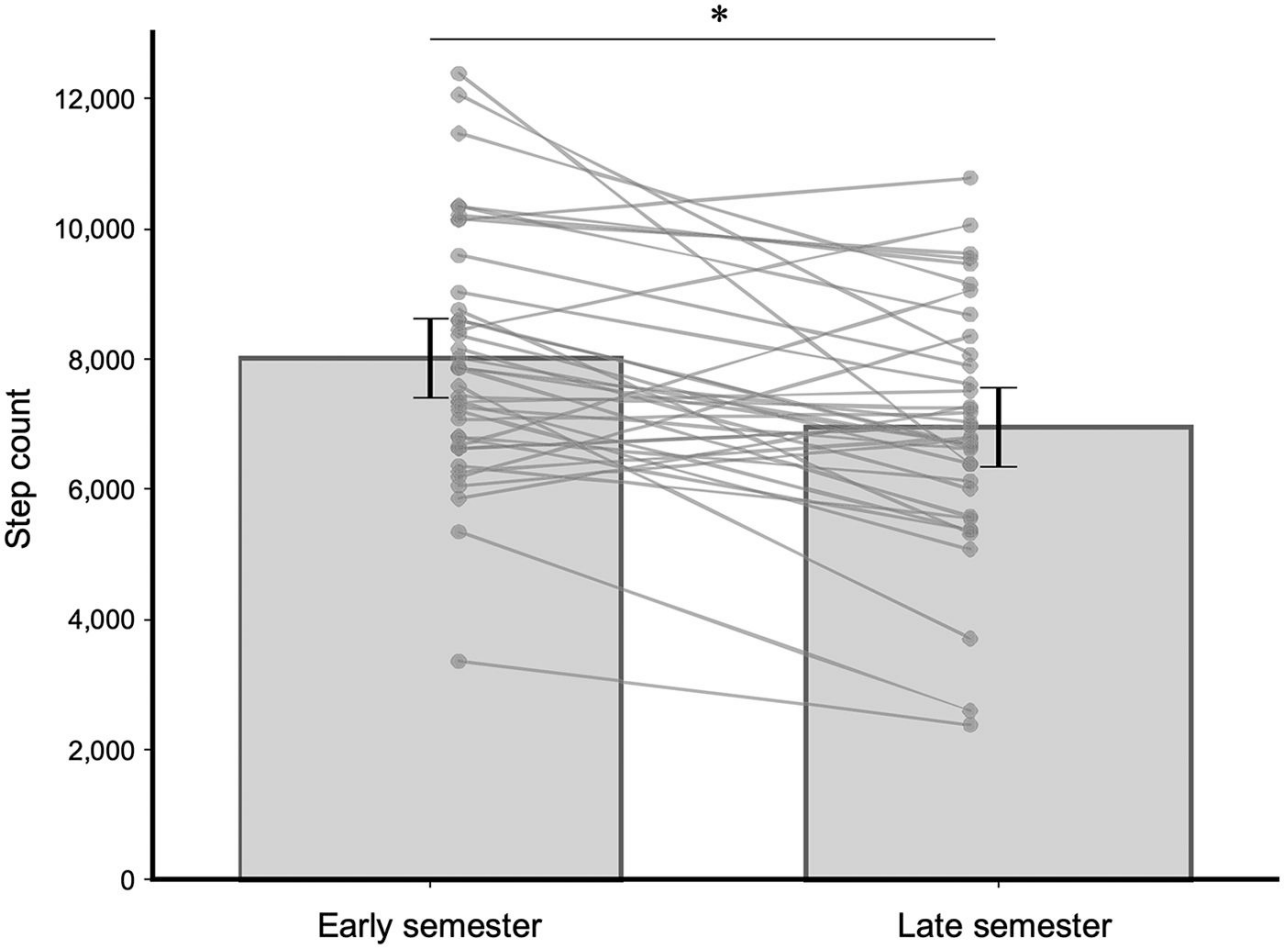
Daily step counts measured early and late in the semester. A paired-samples *t*-test revealed a significant decline in step counts from early to late semester [*n* = 39, *t*(38) = −3.85, *p* < 0.001].

Complete paired step-count data were available for 39 students. A paired-samples *t*-test revealed a significant decrease in step counts from the early to late semester assessment [*t*(38) = −3.85, *p* < 0.001]. On average, participants took 1,057 fewer steps per day toward the end of the semester (95% CI: 501–1,613), with a medium-to-large effect size (Cohen's *d* = −0.62). This downward trend was observed in the majority of students and was reflected in the group means (Figure 3). This finding indicates that objectively measured free-living ambulatory activity did not improve over the semester.

### 3.4 Secondary session-specific affective response during the Mölkky session

**Figure 4:**
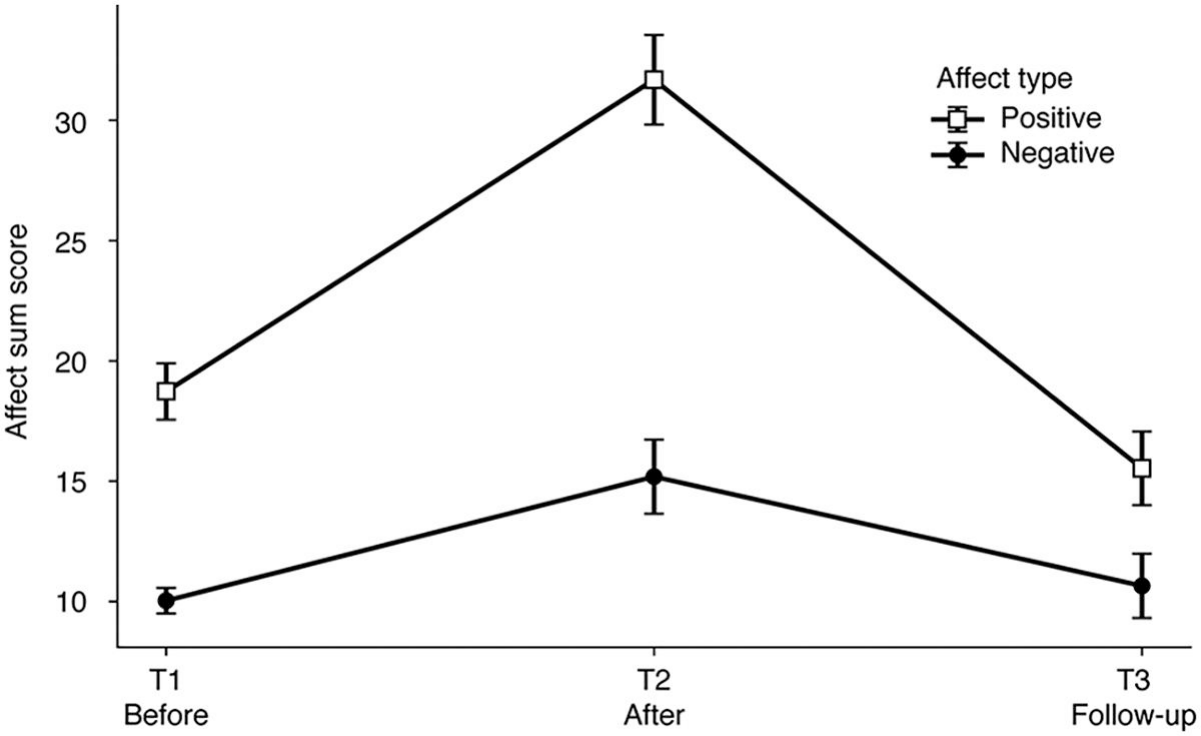
Positive and negative affect scores before and after the group-work (mölkky) session. Scores are shown for participants who completed all three PANAS assessments (*n* = 26). Positive affect increased immediately after the Mölkky session and decreased at follow-up. Negative affect also increased immediately after the session and returned toward baseline at follow-up. Error bars indicate standard errors.

As a secondary session-specific outcome, PANAS responses were analyzed among participants who completed all three assessments (*n* = 26). Positive affect changed significantly across time points [F(2, 50) = 72.29, *p* < 0.001, Figure 4]. Positive affect increased from before the Mölkky session to immediately after the session (mean difference = 12.96, Holm-adjusted *p* < 0.001), but decreased at follow-up compared with both immediately after the session (mean difference = −16.15, Holm-adjusted *p* < 0.001) and baseline (mean difference = −3.19, Holm-adjusted *p* = 0.004).

Negative affect also changed significantly across time points [F(2, 50) = 6.72, *p* = 0.003]. Negative affect increased from before the Mölkky session to immediately after the session (mean difference = 5.15, Holm-adjusted *p* = 0.005), and then decreased at follow-up compared with immediately after the session (mean difference = −4.54, Holm-adjusted *p* = 0.025). Negative affect at follow-up did not differ significantly from baseline (mean difference = 0.62, Holm-adjusted *p* = 0.674).

## 4 Discussion

In this exploratory single-group evaluation, the mixed-effects ordinal model indicated that students were more likely to be classified in higher exercise behavior readiness stages at the post-course assessment than at baseline. Weekly self-rated effort toward self-selected health goals also increased over the semester, and the Mölkky session elicited acute changes in both positive and negative affect among PANAS complete cases. In contrast, objectively measured step counts significantly decreased from early to late semester. Taken together, these findings suggest that the course was associated with changes in exercise behavior readiness and engagement-related indicators, but they do not demonstrate improvement in objectively measured free-living physical activity or causal intervention effectiveness.

The course was designed using SDT as a pedagogical framework, with activities intended to provide opportunities for choice, skill-building, reflection, and social interaction among university students. The observed increases in weekly goal-attainment effort and session-specific positive affect may be compatible with this design rationale (28–30). However, because we did not directly measure SDT-relevant constructs such as perceived autonomy support, autonomy, competence, relatedness, basic psychological need satisfaction, or motivational regulation, the present data cannot identify SDT-based mechanisms. Future studies should include direct measures of these constructs to test whether SDT-related processes mediate changes in exercise behavior readiness or physical activity behavior.

Over four months and 15 sessions, the course was associated with an initial shift in stage classification, particularly among students without an established exercise habit (pre-contemplation/contemplation stages). However, whether similar shifts occurred among already-active students is less clear. Across both stage classification and goal-attainment effort, changes were more pronounced among students with lower baselines, whereas those starting from mid-to-high baselines exhibited smaller gains or occasional regression over the semester (Figures 1, 2). We interpret this pattern along two dimensions.

First, instructional content and activity formats may have offered greater novelty and informational value to lower-baseline students while feeling familiar or redundant those already performing regular PA (31). Although goal setting was individualized to cultivate autonomy within an SDT framework, the incremental behavioral leverage for students who already engaged in routine health goal setting may have been modest—without negating the broader pedagogical value of the course. Second, while the 15 sessions offered a diverse range of activities, we deliberately prioritized competence-building and broad accessibility, with only one session (team handball) providing a distinctly high-intensity competitive stimulus. For students accustomed to vigorous training, the relative physical load may have felt under-dosed (32) and insufficient to satisfy a desire for more demanding exertion (33). More generally, addressing the health needs of cohorts with wide variations in baseline exercise habits is inherently constrained within a single course (34), and verifying durable behavior change within a four-month, 15-session window is similarly limited (35). These considerations suggest that beyond optimizing a single course, program-level, multi-course approaches that serve heterogeneous needs over longer horizons are warranted.

The PANAS assessment provided secondary, session-specific evidence of acute affective responses to the cooperative Mölkky session. In the complete-case analysis, positive affect increased markedly immediately after the session, but negative affect also increased transiently and returned toward baseline at follow-up. This pattern suggests that the session may have elicited heightened affective activation, including both positive engagement and some degree of tension, arousal, or competitive stress. Therefore, the PANAS findings should not be interpreted as evidence that the overall course improved affect, motivation, or exercise behavior. Rather, they indicate that a single cooperative recreational activity can evoke short-term affective responses that warrant more detailed evaluation in future studies. From the perspective of course design, Mölkky remained relevant as an inclusive, low-intensity, socially interactive activity intended to provide opportunities for relatedness-supportive experiences. However, because relatedness and motivational regulations were not directly measured, the present data do not establish an SDT-based mechanism.

Contrary to our original hypothesis, objectively measured step counts significantly decreased from early to late semester. This finding indicates that the course was not associated with improvement in free-living ambulatory activity during the observation period and substantially constrains any claim regarding behavioral improvement. Seasonal heat may have contributed to the decline, particularly because the late-semester monitoring period occurred during hot summer conditions and outdoor activities were limited for safety reasons. Rising temperatures from spring to summer in Japan substantially reduce opportunities for outdoor activity. Tanaka et al. (36) reported a significant seasonal decrease in PA among young women, including university students, in Hyogo Prefecture, adjacent to Osaka, where this study was conducted. Their results showed a step-count reduction from 10,922 ± 2,523 to 9,298 ± 2,570 steps/day from spring to summer, a decline of approximately 1,624 steps/day (Table 4 in Tanaka et al.), which is slightly greater than that observed in our study. This pattern is broadly consistent with the seasonal direction of the present study, in which measurements were conducted in May and July. However, the Tanaka et al. study involved a different sample and observational design, and the present study did not formally model temperature, humidity, academic schedule, or other contextual factors. In line with the Japan Sports Association's Heatstroke Prevention Guidebook for Sports, we avoided outdoor physical activities when the wet bulb globe temperature exceeded 31 °C (37). Consequently, most sessions were conducted indoors after mid-June (Table 1). These circumstances provide a plausible context for the observed decline in step counts during the latter half of the semester. However, this explanation remains speculative because temperature and other environmental factors were not formally modeled and no control group was included. In the future, designing HL courses that consider seasonal variability in temperature, particularly in countries with distinct climatic shifts such as Japan, represents a promising and contextually relevant research direction.

### 4.1 Limitations

This study has several limitations. First, the single-group pre–post design without a control group limits causal inference. One-group pretest–posttest designs are vulnerable to threats to internal validity, including history, maturation, testing effects, regression to the mean, and other contextual influences (38). In the absence of a comparable group of students who did not participate in the course, it is not possible to determine whether the observed within-participant changes were attributable to the course itself or to other factors, such as adaptation to university life, seasonal variation, academic demands, novelty effects, or demand characteristics.

Second, the sample size was small. Although the mixed-effects ordinal model indicated a significant within-participant shift in exercise behavior readiness, the study included only 44 students, and 42 completed the final assessment for the primary outcome. Therefore, the precision and stability of estimates should be confirmed in larger samples.

Third, participants were recruited from a single required credit-bearing course at one university. The course was one of several concurrently offered required course options for first-year students, and students were assigned to one of these courses through the university's lottery-based allocation system. Although this allocation procedure may reduce the likelihood that participants self-selected this specific health literacy course based on prior interest in health, physical activity, or data-based learning, the findings are still based on one cohort, one course, and one institutional context. This limits external validity.

Fourth, although the course was designed to be consistent with SDT principles, we did not directly measure SDT-relevant constructs, such as perceived autonomy support, autonomy, competence, relatedness, basic psychological need satisfaction, or motivational regulation. Therefore, SDT-based mechanisms cannot be inferred from the present data.

Fifth, weekly goal-attainment effort reflected self-rated effort toward self-selected goals, which were heterogeneous and could be modified during the semester. Thus, these scores should be interpreted as indicators of self-regulatory engagement rather than direct measures of standardized behavior change.

Sixth, step counts were measured during only two seven-day periods and may not fully represent free-living physical activity across the entire semester. The observed decline may have been influenced by temporary contextual factors, including weather and academic schedule.

Finally, the PANAS analysis was limited to 26 students who completed all three assessments, which may introduce selection bias. Moreover, affect was assessed during only one Mölkky session. Therefore, these findings should be interpreted as session-specific acute affective responses and should not be generalized to the entire course. Because both positive and negative affect increased immediately after the session, future studies should examine more detailed affective dimensions, including enjoyment, tension, arousal, and perceived competition.

Although the course was framed as a health literacy course, health literacy itself was not directly measured as an outcome. The present study focused on exercise behavior readiness and related process indicators within a health literacy course context, rather than on pre–post changes in students’ health literacy. Therefore, the findings should not be interpreted as evidence that the course improved health literacy. Future studies should include validated health literacy measures when evaluating whether such courses improve health literacy itself or whether changes in health literacy mediate changes in exercise behavior readiness or physical activity behavior.

## 5 Conclusions

In this pragmatic single-group evaluation, participation in an SDT-informed health literacy course was associated with a within-participant shift toward higher exercise behavior readiness and increased self-rated effort toward self-selected health goals. A cooperative Mölkky session elicited acute, session-specific changes in both positive and negative affect. However, objectively measured step counts decreased over the semester, indicating that free-living ambulatory activity did not improve during the observation period. Given the absence of a control group, small sample size, and limited follow-up, these findings should be interpreted as preliminary and exploratory. Future controlled or randomized studies with longer follow-up and direct measures of SDT-related constructs are needed to evaluate the efficacy and mechanisms of curriculum-integrated health literacy courses.

## Data Availability

The datasets presented in this study can be found in online repositories. The names of the repository/repositories and accession number(s) can be found below: https://doi.org/10.6084/m9.figshare.30604385.
