# Supplementary material for: A pragmatic single-group evaluation of a self-determination theory-informed health literacy course for exercise behavior readiness among Japanese university students
Source: Front Sports Act Living. 2026 Jul 15;8:1821452. doi: 10.3389/fspor.2026.1821452 (PMC13416518; doi:10.3389/fspor.2026.1821452)
Supplement: Supplementary file 1 [file Supplementaryfile1.docx]

Supplementary Material 1

# Session Details

## Nordic Walking on Campus

The purpose of this session was to introduce one form of aerobic exercise through Nordic walking practice and allow students to experience the enjoyment of exercising in the natural campus environment. Students first watched an instructional video to understand the basic technique, then practiced pole handling and fundamental walking in an outdoor field. They explored how physical load varied depending on rhythm and pole manipulation. Students then walked freely around the Toyonaka Campus of The University of Osaka for approximately 30 minutes. Initially, their walking was awkward, but as they developed rhythm and pole coordination, smooth and brisk walking emerged. Although all participants were first-time Nordic walkers, the activity accommodated a range of individual fitness levels, resulting in a high degree of satisfaction.

## Blindness Simulation and Sight-Guided Practice

The aim of this session was to achieve multiple learning outcomes through a guided campus walk in which one partner simulated visual impairment. The objectives were as follows:

Physiological learning: To identify one’s position on campus using nonvisual sensory information and memory.

Kinesiological learning: To improve and maintain physical fitness through a walking activity.

Welfare and inclusivity learning: To acquire appropriate guiding techniques and considerate behavior toward individuals with visual impairment and to understand how to ensure safety in real-world environments.

Sociological learning: To identify areas of the campus with insufficient barrier-free accessibility and to consider possible improvements.

Psychological learning: To experience how physical contact with a guide partner can contribute to emotional stability.

Participants worked in pairs. The student simulating visual impairment wore an eye mask and a reflective vest, while the guide was given a campus map indicating four well-known checkpoints. Guides were instructed to lead their partners safely through these checkpoints and return to the starting point. Students simulating visual impairment were asked to recall the route and buildings they passed, relying on nonvisual sensory cues, including auditory (e.g., campus bus engines), olfactory (e.g., cafeteria aromas), somatosensory (e.g., directional changes), and tactile (e.g., hard brick paths vs. soft grass) information.

After learning proper guiding techniques from an instructional video, participants practiced in a secured outdoor area until guides felt confident, and simulated visually impaired partners could walk smoothly without apprehension. Subsequently, the full guidance exercise began. After approximately 40 minutes, all teams successfully completed the task and returned to the starting point. Each student who had simulated visually impairment then marked the recalled route and checkpoints on a map, and the group evaluated the accuracy of memory recall.

## Handball

The objective of this session was to engage students in a relatively high-intensity and complex physical activity, develop sport-specific strategies and tactics, and enhance team cohesion. As approximately half the students had prior handball experience through physical education classes, four teams (11 members each) were organized with balanced skill levels. After practicing individual shooting skills, they completed three-on-three tactical drills, followed by formal seven-on-seven games. Each game lasted six minutes with frequent substitutions to ensure full participation. During play, heart rate (HR) was measured using the Polar Verity Sense (Polar Electro, Finland) and displayed on a tablet as color-coded zones corresponding to percentage of maximum HR. This visualization enabled students to associate subjective fatigue with quantitatively measured physiological load in real time.

## Ankle Taping

This session aimed to develop taping skills for preventing ankle inversion sprains, a fundamental technique in sports medicine. Students first learned the mechanisms of lateral ankle sprain—one of the most common sports injuries—involving calcaneal inversion beneath the medial malleolus and consequent stress on the anterior talofibular ligament (ATF). They palpated the ATF to locate it and understand the correct taping pathway. In pairs, students practiced taping on each other’s ankles using the following techniques: underwrap, anchor, stirrup (to restrict calcaneal inversion), horseshoe, and figure-of-eight. The practice was repeated across two sessions. Although they did not reach the level required for competitive use, all students achieved a satisfactory mastery of the fundamental skills. The most complete taping results were photographed with smartphones and uploaded to an educational cloud system for evaluation.

## Campus Quiz Orienteering

The purpose of this session was to increase physical activity through a large-scale campus orienteering event while fostering teamwork through friendly competition. Eight teams of approximately five members each received a campus map and a list of 20 quiz-style questions related to specific locations within the Toyonaka Campus. Each question required on-site observation to answer, and questions were distributed throughout the campus. Eight distinct question sets were prepared so that each team faced different challenges. Teams could divide tasks among members, but all had to return to the goal together within the 60-minute time limit. Scoring rules were as follows: six points per correct answer, plus additional points equal to the remaining minutes if all members returned within the limit. If a team exceeded the limit, points equal to the overtime minutes were deducted. This scoring system encouraged both physical and cognitive strategy, as success depended on team coordination, spatial planning, and time management. During the orienteering, HR and acceleration (ACC) were monitored with the Polar Verity Sense.

## Grip Strength Grading Experiment

The goal of this session was to make visible the relationship between subjective motor output intensity and actual physical output through a classical psychophysical task in motor control research: grip strength grading. In pairs, participants alternated between experimenter and participant roles, using an analogue handgrip dynamometer (T.K.K.5401, SANKA Co., Ltd., Japan). The participant first performed two maximal voluntary contractions (eyes open) to establish a reference perception of exertion level. Then, they were asked to reproduce 20%, 40%, 60%, 80%, and 100% of maximal force in random order, without feedback. The test was performed twice for each hand. After completing all trials, roles were switched. Results were immediately entered into Google Forms (Google LLC, USA) for real-time aggregation, visualizing discrepancies between subjective and actual outputs (Figure S1-1). Students also examined whether the data followed Stevens’ power law and discussed why the measured force tended to exceed target values at lower levels but fall below them at higher levels.

**Supplementary Figure S1-1.** Relationship between subjectively targeted and objectively measured grip strength.

## Mölkky

In this session, participants cultivated a sense of camaraderie through team-based participation in Mölkky while being introduced to a lifelong recreational sport that can be enjoyed regardless of physical fitness. Mölkky, developed by Tuoterengas Ltd. in 1996 and based on the traditional Finnish game *kyykkä*, involves throwing a wooden stick (*mölkky*) at numbered pins (skittles) to score points. It has become increasingly popular in Japan due to its accessibility. The session was conducted over two consecutive weeks. In the second week, the psychological effects of the group-based activity were assessed using the Positive and Negative Affect Schedule (PANAS) administered before the session, immediately after, and again after a subsequent lecture class. Eight teams of approximately five members each competed in five matches per team, with each match lasting approximately 10 minutes. Regardless of gender or fitness level, all students showed strong engagement and enjoyment throughout the games. PANAS results (Figure 4 in the main text) indicated that the Mölkky session elicited acute, session-specific changes in both positive and negative affect.

## Visualization of Physical Load Across Sessions

To quantify the physical load in each session, time-series HR and ACC data from the Polar Verity Sense were summarized and visualized. Each student’s physical load in a given session was represented as a point on a two-dimensional plane defined by HR and ACC indices. The HR time series was sorted in descending order, and the third quartile value was extracted as a representative measure. For the ACC data, following the method of Marutani et al. (1), the norm of the ACC signal was computed, and its histogram was fitted with a Gaussian mixture model. The high-magnitude component of this model was then used as the representative value.

Each student’s physical load per session was thus expressed as a two-dimensional vector. Figure S1-2 shows a scatterplot, where each marker corresponds to an individual student. For each session, a 95% confidence ellipse was also plotted. Classroom-based activities such as grip strength grading practice, Mölkky, and pickleball (conducted in another class) were relatively low in intensity, whereas basketball (conducted in another class) and handball involved higher intensity levels. Orienteering exhibited a horizontally elongated confidence ellipse, indicating greater interindividual variability in physical load.

These visualized data were presented to students during the Data Review Session as feedback. Discussion confirmed that the amount of physical activity differed by activity type and emphasized the importance of selecting appropriate physical activities according to individual fitness levels.


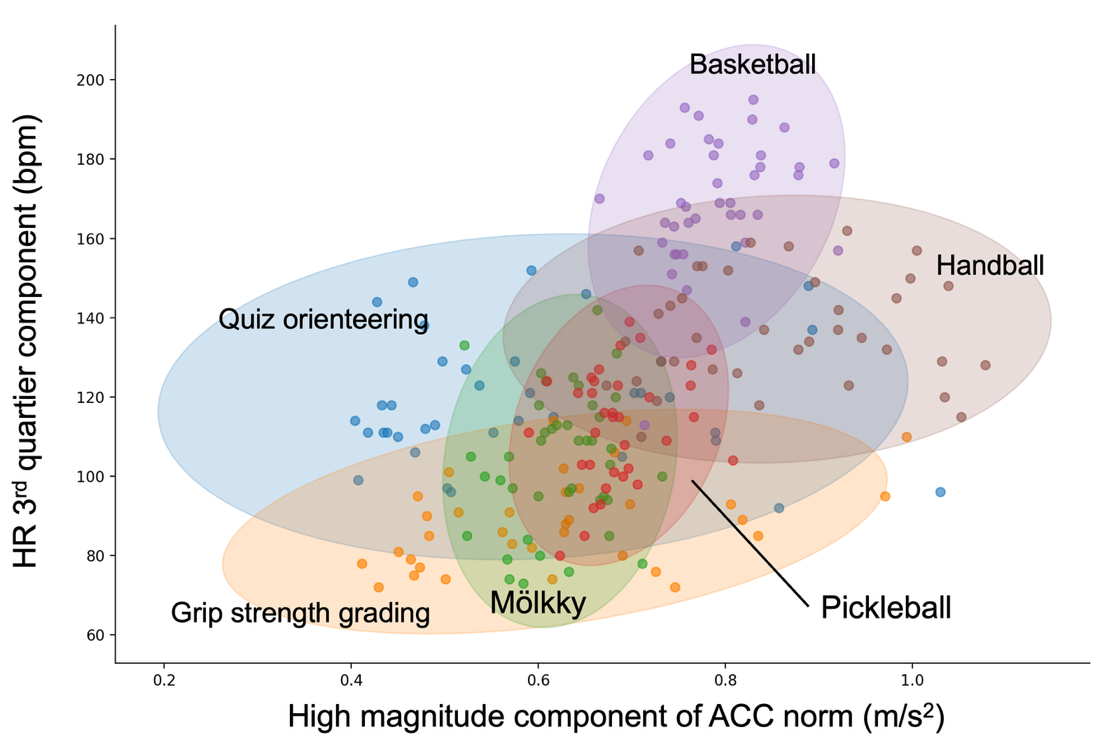


**Supplementary Figure S1-2.** Scatterplot of heart rate and acceleration indices during each session.

**References**

1. Marutani Y, Konda S, Ogasawara I, Yamasaki K, Yokoyama T, Maeshima E, et al. Gaussian mixture modeling of acceleration-derived signal for monitoring external physical load of tennis player. Front Physiol (2023) 14:1161182. doi: 10.3389/fphys.2023.1161182
