# Supplementary material for: A pragmatic single-group evaluation of a self-determination theory-informed health literacy course for exercise behavior readiness among Japanese university students
Source: Front Sports Act Living. 2026 Jul 15;8:1821452. doi: 10.3389/fspor.2026.1821452 (PMC13416518; doi:10.3389/fspor.2026.1821452)
Supplement: Supplementary file 2 [file Supplementaryfile2.docx]

Supplementary Material 2

# Table S2. Goal setting and updates

| ID | 22-Apr | 7-May | 13-May | 20-May | 27-May | 3-Jun | 10-Jun | 17-Jun | 24-Jun | 8-Jul | 15-Jul | 22-Jul | Goal update count | Type of goal |
| --- | --- | --- | --- | --- | --- | --- | --- | --- | --- | --- | --- | --- | --- | --- |
| 01 | 8,000 steps/day |  |  |  |  |  |  |  |  |  |  |  | 0 | PA/Ex-related |
| 02 | Exercise twice per week; join a club or walk/run if not. |  |  |  | Do strength training twice a week with running. |  |  |  |  |  |  |  | 1 | PA/Ex-related |
| 03 | Lose weight and fat; avoid fatty food; do daily strength training. |  |  |  |  |  |  |  |  |  |  |  | 0 | Body composition/weight-related, PA/Ex-related |
| 04 | Regain strength with daily upper-body training and some running. |  |  |  |  |  |  |  |  |  |  |  | 0 | PA/Ex-related |
| 05 | Make muscle training a daily habit. |  |  |  |  |  |  |  |  |  |  |  | 0 | PA/Ex-related |
| 06 | Go to the gym 5 times per week even with a part-time job. |  | Do aerobic exercise, too. | Add cardio to training. | Don’t skip cardio! | Keep exercising during exams. | Go to the gym after exams. | Don’t skip gym! |  |  |  |  | 3 (Actually) | Social/general health |
| 07 | Walk to home instead of taking the bus several times a week. |  |  |  |  |  |  |  |  |  |  |  | 0 | PA/Ex-related |
| 08 | Start weekly push-ups and aim to match the abilities of a fit friend. |  |  |  |  |  |  |  |  |  |  |  | 0 | Social/general health |
| 09 | Do muscle training or running 3 times per week. |  | Increased to 4 times weekly with friends. |  |  |  |  |  |  |  |  | Achieved through club and personal workouts. | 2 | PA/Ex-related |
| 10 | Play soccer weekly to regain strength and gain muscle. |  |  |  |  |  |  |  |  |  |  |  | 0 | PA/Ex-related |
| 11 | Swim 30 minutes once per week. |  |  |  |  |  |  |  |  |  |  |  | 0 | PA/Ex-related |
| 12 | Use stairs instead of escalators once per day. |  |  |  |  |  |  |  |  |  |  |  | 0 | PA/Ex-related |
| 13 | Increase arm size by 2 cm by doing daily push-ups. |  |  |  |  |  |  |  |  |  |  |  | 0 | PA/Ex-related |
| 14 | Start a new sport; exercise weekly. | Exercise twice weekly. |  |  |  |  |  |  |  |  |  |  | 2 | PA/Ex-related |
| 15 | Join a club and exercise weekly; walk with friends. |  |  |  |  |  |  |  |  |  |  |  | 0 | PA/Ex-related |
| 16 | Regain 70% endurance by running 20 km weekly; do daily bodyweight training. |  |  |  |  |  |  |  |  |  |  |  | 0 | PA/Ex-related |
| 17 | Do daily stretching to reach 90° splits and prevent injury. |  |  |  |  |  |  |  |  |  |  |  | 0 | PA/Ex-related |
| 18 | Exercise 1 hour once per week. |  |  |  |  |  |  |  |  |  |  |  | 0 | PA/Ex-related |
| 19 | Lose 3 kg by walking daily and training 3 times weekly. |  |  |  |  |  |  |  |  |  |  |  | 0 | Body composition/weight-related, PA/Ex-related |
| 20 | Use stairs instead of elevators. | Continue same goal. | Walk 50,000+ steps weekly. | Walk 60,000+ steps weekly. |  |  | Eat healthy. |  | Walk 10,000 steps daily. | Walk 12,000 steps daily. |  |  | 4 | Diet/nutrition-related, PA/Ex-related |
| 21 | Do intense exercise at least once per week. |  |  |  |  |  |  |  |  |  |  |  | 0 | PA/Ex-related |
| 22 | Take a walk once per week in addition to commute-related walking. |  |  |  |  |  |  |  |  |  |  |  | 0 | PA/Ex-related |
| 23 | Join a weekly club; learn a new sport; do light workouts on off days. |  |  |  |  |  |  |  |  |  |  |  | 0 | PA/Ex-related |
| 24 | Do daily stretching after bathing; avoid using the elevator. |  |  |  |  |  | Go to gym weekly; stretch daily. |  |  |  |  |  | 1 | Social/general health |
| 25 | Gain 2 kg of muscle lost during exams. |  |  |  |  |  |  |  |  |  |  |  | 0 | Body composition/weight-related |
| 26 | Exercise twice per week for 1+ hour; mainly basketball. |  |  |  |  |  |  | Ate a lot but tried to stay active. |  |  | Start home training with brother; keep routine during vacation. |  | 2 | Diet/nutrition-related, PA/Ex-related |
| 27 | Eat breakfast daily and include vegetables at least once per day. |  |  |  |  |  |  |  |  |  |  |  | 0 | Diet/nutrition-related |
| 28 | Exercise daily to stay fit; do muscle training every day. |  |  |  |  |  |  |  |  |  |  |  | 0 | PA/Ex-related |
| 29 | Play tennis twice weekly; do hot yoga if possible. |  |  |  |  |  |  | Eat balanced meals; drink 2 L water daily. |  |  |  |  | 1 | Diet/nutrition-related, PA/Ex-related |
| 30 | Make friends, walk around campus, and play e-sports. |  |  | Exercise daily to lose weight. |  |  |  |  |  |  |  |  | 1 | Social/general health, PA/Ex-related, Body composition/weight-related |
| 31 | Avoid daily sweets; do stretching and muscle training to reduce fat. |  |  |  |  |  |  |  |  |  |  |  |  | Diet/nutrition-related, PA/Ex-related, Body composition/weight-related |
| 32 | Dance for health at least twice per week using YouTube. |  |  |  |  |  |  |  |  |  |  |  |  | PA/Ex-related |
| 33 | Join a gym to build muscle. |  |  | Go to gym 3 times per week. | Bench press 85 kg. |  |  |  |  |  |  |  | 2 | PA/Ex-related |
| 34 | Do workout daily and record it on calendar. |  |  |  |  |  |  |  |  |  |  |  |  | PA/Ex-related |
| 35 | Exercise daily, like walking, to sleep naturally by 23:30. |  |  |  |  |  |  |  |  | Do daily stretching to prevent injury. | Include learned exercises in daily life. |  | 2 | PA/Ex-related, Sleep/recovery-related |
| 36 | Gain 3 kg of muscle; eat well and do muscle training 4 times per week. |  |  |  |  |  |  |  |  |  |  |  | 0 | Diet/nutrition-related, PA/exercise-related, Body composition/weight-related |
| 37 | Eat homemade meals; walk or do light exercise daily. |  |  |  |  |  |  |  |  |  |  |  | 0 | Diet/nutrition-related, PA/exercise-related |
| 38 | Go outside and move body on Sundays, too. |  |  |  |  |  |  |  |  |  | Continue activity on holidays. |  | 0 | Social/general health |
| 39 | Join a sports club to exercise weekly and build stamina. |  |  |  |  | Lose 2 kg starting today! |  |  |  |  |  |  | 0 | Social/general health, Body composition/weight-related |
| 40 | Lose 1.5 kg and tone stomach after exam weight gain. |  |  |  |  |  |  | Lose another 1.5 kg; do ab workouts. |  |  |  |  | 0 | Body composition/weight-related |
| 41 | Run 20 minutes weekly to improve fitness. |  |  |  |  |  |  |  |  |  |  |  | 0 | PA/Ex-related |
| 42 | Use bicycle instead of bus/train (ride 5 times per week). |  |  | Do daily training and stretching (15 min). |  |  |  |  |  |  |  |  | 1 | PA/Ex-related |
| 43 | Walk 7,000+ steps on weekdays by using farther bus stop. |  |  |  |  |  | Walk 8,000+ steps daily, including commute. |  |  |  |  |  | 1 | PA/Ex-related |
| 44 | Exercise 30+ minutes weekly via club; jog or walk if absent. |  |  |  |  |  |  |  |  |  |  |  | 0 | PA/Ex-related |

Note: PA/Ex-related: Physical activity/Exercise related.

Note: The “Type of goal” column shows the descriptive content categories of each student’s self-set goal. For the sensitivity analysis reported in the main text, participants whose goals included both PA/Ex-related and non-PA/Ex-related components were conservatively classified into the non-PA/Ex-related group.
