# Supplementary material for: A pragmatic single-group evaluation of a self-determination theory-informed health literacy course for exercise behavior readiness among Japanese university students
Source: Front Sports Act Living. 2026 Jul 15;8:1821452. doi: 10.3389/fspor.2026.1821452 (PMC13416518; doi:10.3389/fspor.2026.1821452)
Supplement: Supplementary file 3 [file Supplementaryfile3.docx]

Supplementary Material 3

# Internal consistency of the Japanese Positive and Negative Affect Schedule (PANAS)

Internal consistency of the Japanese PANAS was evaluated at each time point using Cronbach’s α, calculated as:

,

where *k* = 16 is the number of items, *s^2^_i_* is the variance of each item, and *s^2^_T_* is the variance of the total score. All three time points yielded high internal consistency, indicating stable reliability across measurements (Table S3).

**Table S3. Internal consistency**

| Time point | Cronbach’s α | Interpretation |
| --- | --- | --- |
| T1: Before Mölkky session | 0.86 | Sufficiently high consistency |
| T2: After Mölkky session | 0.88 | Very high consistency |
| T3: Follow-up | 0.86 | Sufficiently high consistency |
